# The central nervous system of Oweniidae (Annelida) and its implications for the structure of the ancestral annelid brain

**DOI:** 10.1186/s12983-019-0305-1

**Published:** 2019-03-12

**Authors:** Patrick Beckers, Conrad Helm, Günter Purschke, Katrine Worsaae, Pat Hutchings, Thomas Bartolomaeus

**Affiliations:** 1 Institute of Evolutionary Biology, University of Bonn, 53121 Bonn, Germany; 2 Johann-Friedrich-Blumenbach Institute for Zoology & Anthropology Animal Evolution and Biodiversity, University of Göttingen, 37073 Göttingen, Germany; 3 Department of Developmental Biology and Zoology, University of Osnabrück, 49069 Osnabrück, Germany; 4 Department of Biology, University of Copenhagen, 2100 Copenhagen, Denmark; 5 Australian Museum Research Institute, Australian Museum, Sydney, NSW 2010 Australia; 6 Department of Biological Sciences, Macquarie University, North Ryde, 2109 Australia

**Keywords:** Neuroanatomy, Brain, Central nervous system, Spiralia, Glia, Ultrastructure, Annelida

## Abstract

**Background:**

Recent phylogenomic analyses congruently reveal a basal clade which consists of Oweniidae and Mageloniidae as sister group to the remaining Annelida. These results indicate that the last common ancestor of Annelida was a tube-dwelling organism. They also challenge traditional evolutionary hypotheses of different organ systems, among them the nervous system. In textbooks the central nervous system is described as consisting of a ganglionic ventral nervous system and a dorsally located brain with different tracts that connect certain parts of the brain to each other. Only limited information on the fine structure, however, is available for Oweniidae, which constitute the sister group (possibly together with Magelonidae) to all remaining annelids.

**Results:**

The brain of Oweniidae is ring- shaped and basiepidermal. Ganglia, higher brain centers or complex sensory organs do not exist; instead the central nervous system is medullary. Posterior to the brain the ventral medullary cord arises directly from the ventral region of the brain in *Myriowenia* sp. while in *Owenia fusiformis* two medullary cords arise perpendicular to the brain ring, extend caudally and fuse posterior. The central nervous system is composed of a central neuropil and surrounding somata of the neurons. According to ultrastructural and histological data only one type of neuron is present in the central nervous system.

**Conclusion:**

The central nervous system of Oweniidae is the simplest in terms of enlargement of the dorsal part of the brain and neuron distribution found among Annelida. Our investigation suggests that neither ganglia nor commissures inside the brain neuropil or clusters of polymorphic neurons were present in the annelid stem species. These structures evolved later within Annelida, most likely in the stem lineage of Amphinomidae, Sipuncula and Pleistoannelida. Palps were supposedly present in the last common ancestor of annelids and innervated by two nerves originating in the dorsal part of the brain. A broader comparison with species of each major spiralian clade shows the medullary nervous system to be a common feature and thus possibly representing the ancestral state of the spiralian nervous system. Moreover, ganglia and clusters of polymorphic neurons seemingly evolved independently in the compared taxa of Spiralia and Annelida.

**Electronic supplementary material:**

The online version of this article (10.1186/s12983-019-0305-1) contains supplementary material, which is available to authorized users.

## Background

The nervous system of annelids has been intensely studied by several authors in the late 19th and early twentieth century, but most comparative investigations were conducted by Orrhage in the last half of the twentieth century [1, 2] for references]. In general, the central nervous system (*cns*) of Annelida is composed of a ventral nerve cord and a prostomial brain. It consists of a central neuropil- (or fibre-) core surrounded by neuronal somata. Besides neurons, glial cells are present and may form a prominent layer around the neuropil and somata [2–4]. All annelids investigated thus far possess an anterior concentration of neuropil and somata receiving most of the sensory input which is generally referred to as the brain [5]. Usually it is located within the prostomium, but may be shifted to a more posterior position in taxa possessing a comparatively small prostomium such as observed in many burrowing taxa, like Clitellata [2, 3]. The annelid brain was historically described as consisting of different parts, the fore-, mid- and hindbrain. The value of this terminology was already doubted by Bullock and Horridge [3], since in most annelids these divisions are hardly or not discernible at all, especially in sedentary species [2, 3]. Higher brain centers like mushroom bodies and glomerular neuropil are present in several errant taxa [2–4, 6–8], but are missing in sedentary species. Several nerves may branch off the brain, innervating the prostomium, the anterior head appendages including antennae, palps and tentacular cirri as well as the stomatogastric system innervating the gut [1–3 for review]. The brain also gives rise to nerves or neurite bundles which are connected to the remaining, ventrally located *cns*, best known as a subepidermal, ganglionic rope-ladder like nervous system from textbook schemes. Until recently, it was always supposed to represent the ancestral state in Annelida [3, 9, 10], despite the fact that the ventral nervous system of several annelid species does not show this structure (see comments in [2, 11]). A recent comparative analysis of species from all major clades within Annelida prompted the hypothesis, that the last common ancestor most likely did not possess such a ventral ganglionic nervous system, but rather a ventral medullary cord in a basiepidermal position [11]. Annelids usually possess a variety of anteriorly located sensory structures among which the nuchal organ is often regarded to represent the most important one [2, 3, 12, 13]. Absence or presence, shape and structure of this presumably olfactory organ was used as a morphological character for phylogenetic analyses and considered to have evolved in the stem lineage of annelids [3, 9, 14]. First doubts of this were presented in the first phylogenomic analyses, which placed those taxa lacking nuchal organs at deep nodes of the annelid tree [15–22]. Other prominent ciliary structures exhibited by certain annelid taxa are ciliated grooves or papillae between neuro- and notopodia called lateral organs. Thought to be an additional sensory structure, the lateral organs usually consist of uniciliated penetrative sensory cells, which are arranged in specific patterns [2, 12, 23]. Nevertheless, so far their presence in representatives of these basally branching taxa within Annelida has not been shown.

The homology of the different head appendages of annelids is still controversial. Traditionally these appendages are called palps, antennae or tentacles and have a sensory and additionally sometimes a feeding function [24, 25]. The appendages are innervated from different tracts inside or outside the brain proper; these innervation patterns were used to homologize the different head appendages as palps or antennae albeit abandoning the term tentacles or using it as a neutral term without any homology implications. The number and position of these various tracts emanating from the ventral and dorsal roots of the circumesophageal connectives and their commissures in the brain have been compared across a broad range of annelid taxa [1, 26, 27], although their evolutionary history and phylogenetic significance within Annelida have never been properly tested. Despite this fact, Müller [28] proposed a generalized scheme of the anterior central nervous system of Annelida. This comprises a dorsally located brain with two dorsal and two ventral main commissures inside the brain neuropil, connected via two lateral circumesophageal connectives to the ventral cord [28].

However, as stressed by Orrhage [26] this scheme did not imply to represent the structure of the last common ancestor of Annelida. Generally, considerations on the ancestral structure of the annelid brain were not phylogeny based, since relationships within Annelida have been debated for a long time and the structure of the nervous system had not been investigated in details for many key taxa. It also remained controversial whether or not the predicted life style of the annelid ancestor was tube-dwelling and largely sedentary or free living and errant [29–31]. Nevertheless, morphology based cladistics analyses [14] and subsequent modifications [9] became widely used and accepted. Until recently phylogenetic analyses using single or just a few genes did not confirm previous morphological cladistics analyses [9, 14] but were unable to provide robust alternatives [32]. During the last decade phylogenomic approaches were applied to infer the relationships among segmented worms [15–18, 20–22]. Since the first analysis has been published, these studies congruently show that Oweniidae and Magelonidae form the sister group to the remaining Annelida, either as a grade or a clade [11, 15, 18, 20, 21]. Since both taxa are sedentary and tube-dwelling, like the subsequently branching annelid groups Apistobranchidae, Psammodrilidae and Chaetopteridae as well as Sipuncula, the question on the primary annelid life style has been solved now and likewise allowing a re-evaluation of the annelid nervous system. The evolution of the ventral cord has been addressed in a recent study by Helm et al., including new data on the ventral *cns* of previous mentioned annelids but not considering the detailed structure of the brain [11]. Immunochemical studies were conducted on the development of the nervous system of *Owenia fusiformis* as well as for the adult morphology of *Galathowenia* [33, 34] and gene expression patterns were investigated in a recent study [35]. While the brain of basally positioned taxa like Magelonidae and Apistobranchidae was studied with histological methods [36, 37] detailed histological and ultrastructural information on neuron morphology and distribution, sensory structures, commissures inside the brain, glial cells as well as innervation pattern of palps was not available for Oweniidae [34, 38, 39]. In order to fill this gap, we studied the anterior nervous system of two oweniid species, *Owenia fusiformis* Delle Chiaje, 1844 and a thus far undescribed species of *Myriowenia sp.* (see [36]), with a comparative approach including serial sectioning and different histological staining techniques as well as immunohistochemistry, μCT, SEM, serial semithin-sectioning and transmission electron microscopy (TEM).

During our survey on the nervous system of annelids we were also able to re-investigate some of the original sections Orrhage made of *Magelona papillicornis* (see [31]) and *Owenia fusiformis* (unpublished) with different staining techniques. These allowed us to better compare and discuss our new data on Oweniidae relative to his previous interpretations on the anatomy of the annelid nervous system, yet now focusing on the origin of the annelid brain.

## Results

### General anatomy

**Fig. 1:**
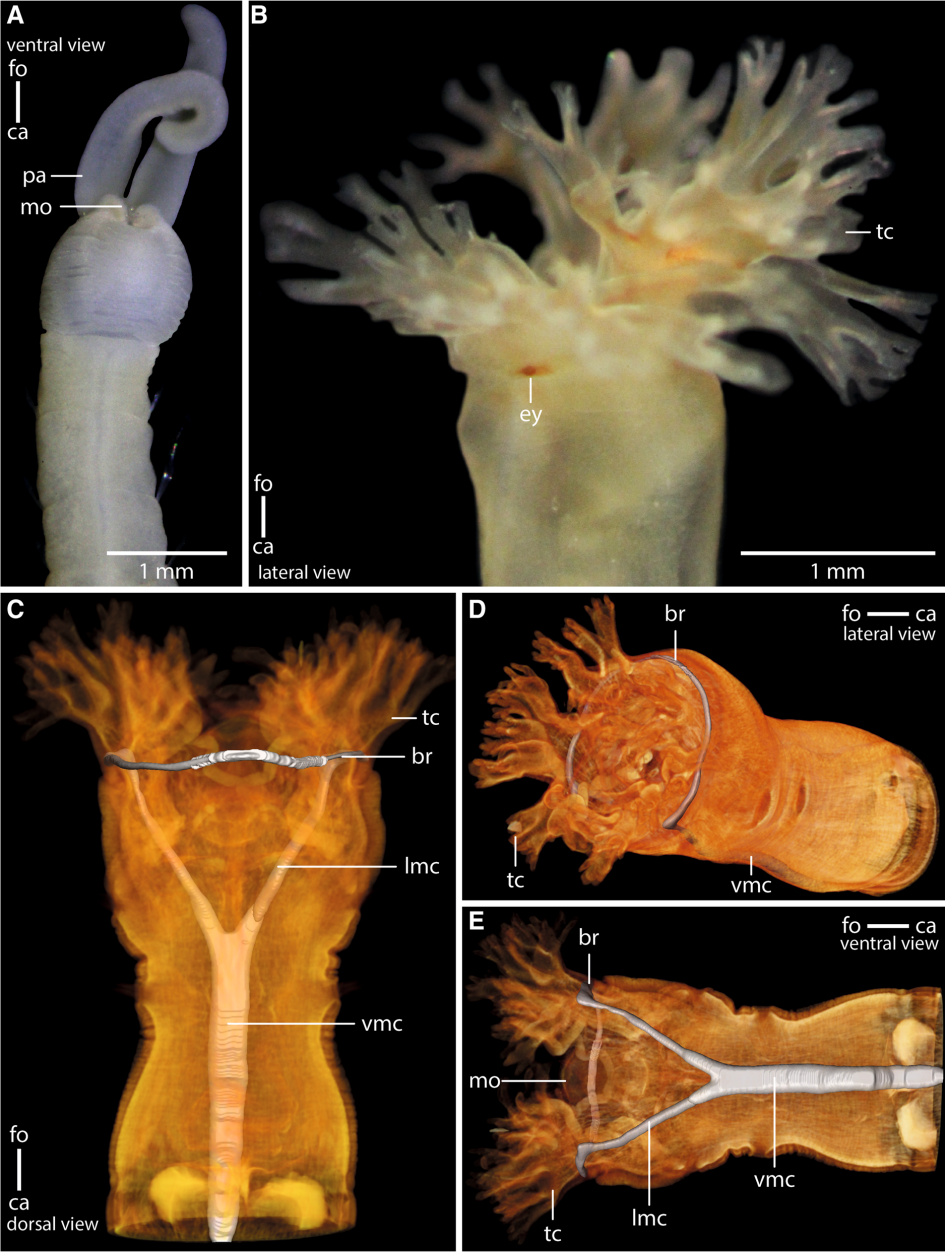
Anterior parts of preserved (7% Formalin) specimen of **a**: *Myriowenia sp*. *pa*: palp. Dorsal view. *ca*: caudal; *fo*: frontal. **b**: Living specimen of *Owenia fusiformis. ey:* eye spot; *tc*: tentacular crown. Lateral view. **c**-**e**: μCt volume rendering and 3D-reconstruction of the nervous system of *Owenia fusiformis* (neuropil only: *grey*), non- segmental commissures not shown (see Fig. 5a). **c**: The ventral medullary cord (*vmc*) arises from paired, perpendicular to the brain ring (*br*) arranged lateral medullary cords (*lmc*) which fuse ventrally. **d**: the brain (*br*) is circular. *tc*: tentacle crown; *vmc*: ventral medullary cord. **e**: the brain (*br*) surrounds the mouth opening (*mo*). *lmc*: lateral medullary cord; *vmc*: ventral medullary cord; *tc*: tentacle crown.

*Myriowenia* sp. and *Owenia fusiformis* differ in the structure of the head appendages and the structure of the anterior intestinal system (Fig. 1). In *Myriowenia* sp., a pair of palps transports food particles into the fronto-ventrally located mouth (Fig. 1a). In *O. fusiformis* the food is grabbed by the large terminal tentacular crown and forced into the terminal, funnel shaped mouth (Fig. 1b-e).

**Fig. 2:**
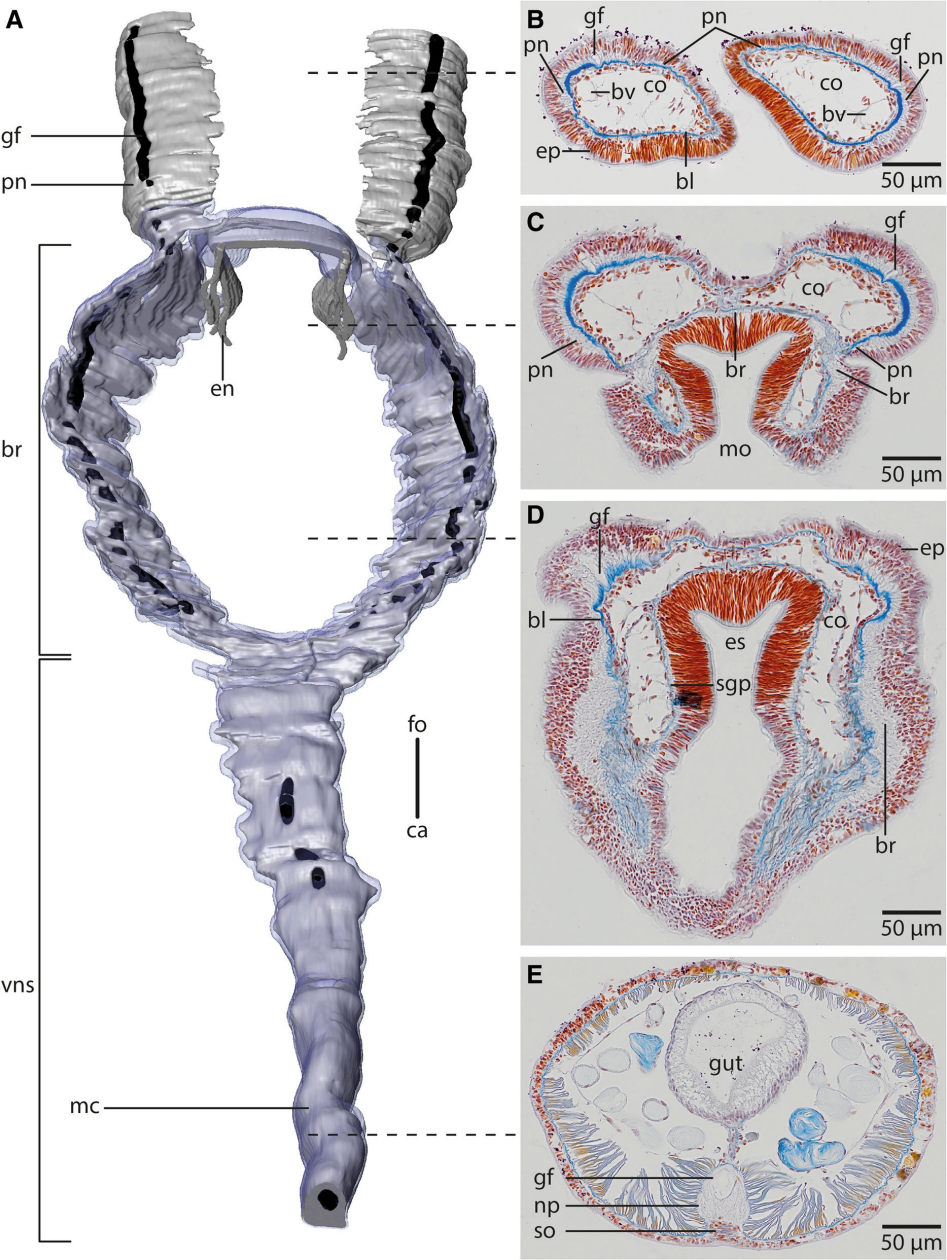
*Myriowenia sp*. **a**: 3D-reconstruction of anterior central nervous system, dorsal view; *grey*: neuropil; *black*: giant fibre; *bright blue*: somata of nerve cells. **b**-**e**: histological cross sections (5 μm), Azan staining, frontal (**b**) to caudal (**e**). **a**: The central nervous system (*cns*), of *Myriowenia sp*. is composed of a ring- shaped brain (*br*). The palp nerve (*pn*) is connected to the brain frontally; caudally the brain gives rise to the ventral medullary cord (*mc*). A giant fibre (*gf*, black) can be traced to the tips of the palps and along the entire medullary cord. The stomatogastric system is innervated by two esophageal nerves (*en*) which arise from the dorso-lateral part of the brain. *ca*: caudal; *fo*: frontal; *vns*: ventral nervous system. **b**: the paired palps contain a palp nerve (*pn*), which is more prominent close to the blood vessels (*bv*). The paired giant fibres (*gf*) can be traced to the tip of the palps. *bl*: basal lamina; *co*: coelom; *ep*: epidermis. **c**: the palp nerve (*pn*) is connected to the brain (*br*) by two nerves (*pn*), of which the more prominent one arises in the dorso- lateral parts of the brain. The stomatogastric plexus (*sgp*) is innervated by a nerve which arises from the inner margins of the dorsal part of the brain. *co*: coelom; *mo*: mouth opening; **d**: the brain (*br*) is located inside the epidermis (*ep*) and extends along the outer margins of the animal. *bl*: basal lamina; *co*: coelom; *es*: esophagus; *sgp*: stomatogastric plexus. **e**: the ventral medullary cord is located inside an epidermal invagination. The giant fibre (*gf*) is enlarged and located dorsally to the neuropil (*np*) of the medullary cord, while somata (*so*) are located ventrally to the latter

**Fig. 3:**
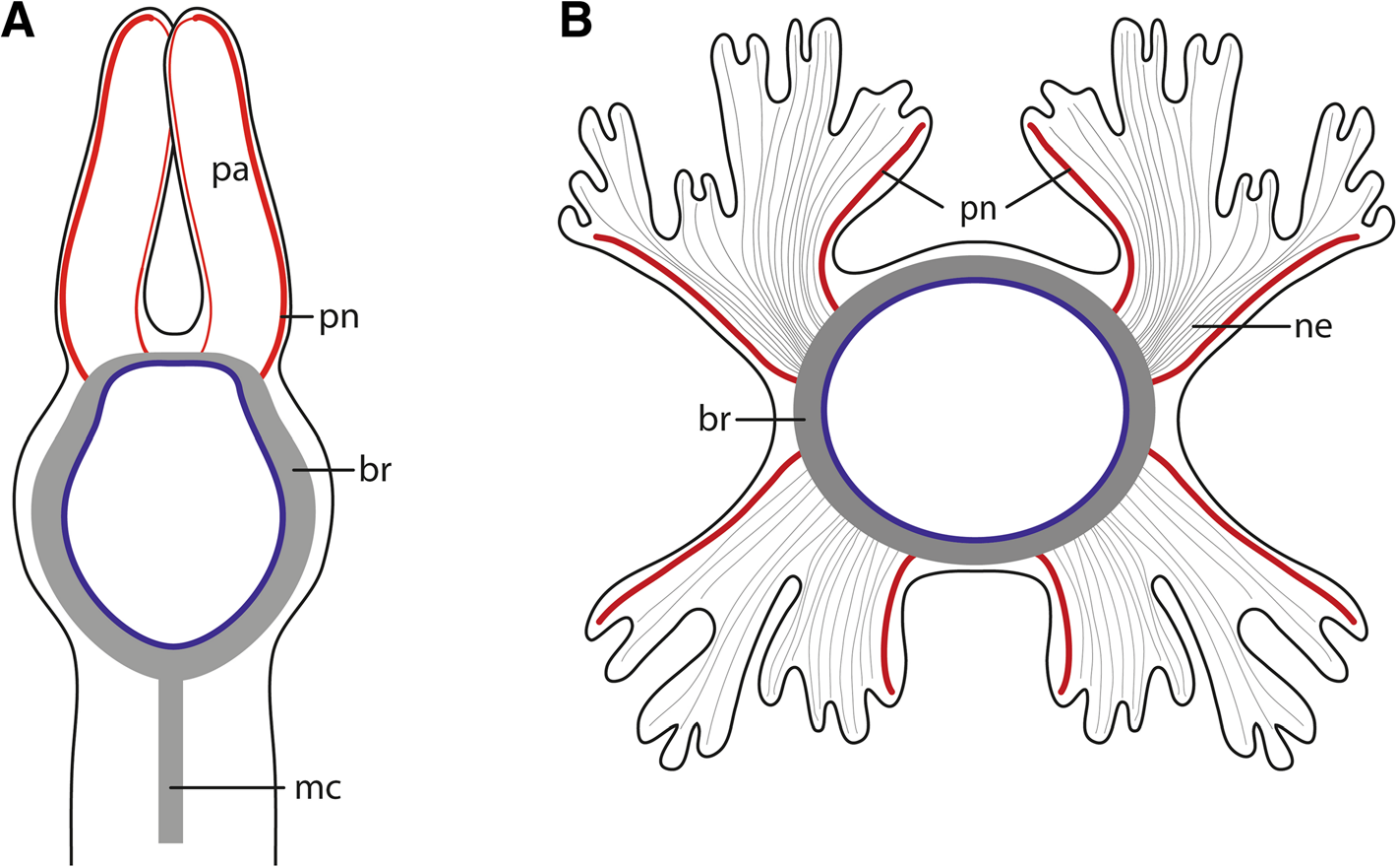
Schematic drawing of the innervation of head appendages. **a**: *Myriowenia* sp.; dorsal view. **b**: *Owenia fusiformis* anterior view, dorsal side up. **a**: Palps (*pa*) of *Myriowenia* sp. are innervated by two nerves (*pn,* red) originating in the dorsal part of the brain ring (*br,* grey). The lateral palp nerve (*pn*) is more prominent than the inner one. *mc*: medullary cord. **b**: Each main branch of the tentacle crown is innervated by two lateral located main nerves (*pn*). The remaining tissue is innervated by fine neurites (*ne*). *blue*: basal lamina of epidermis; *grey* neuropil of brain

**Fig. 4:**
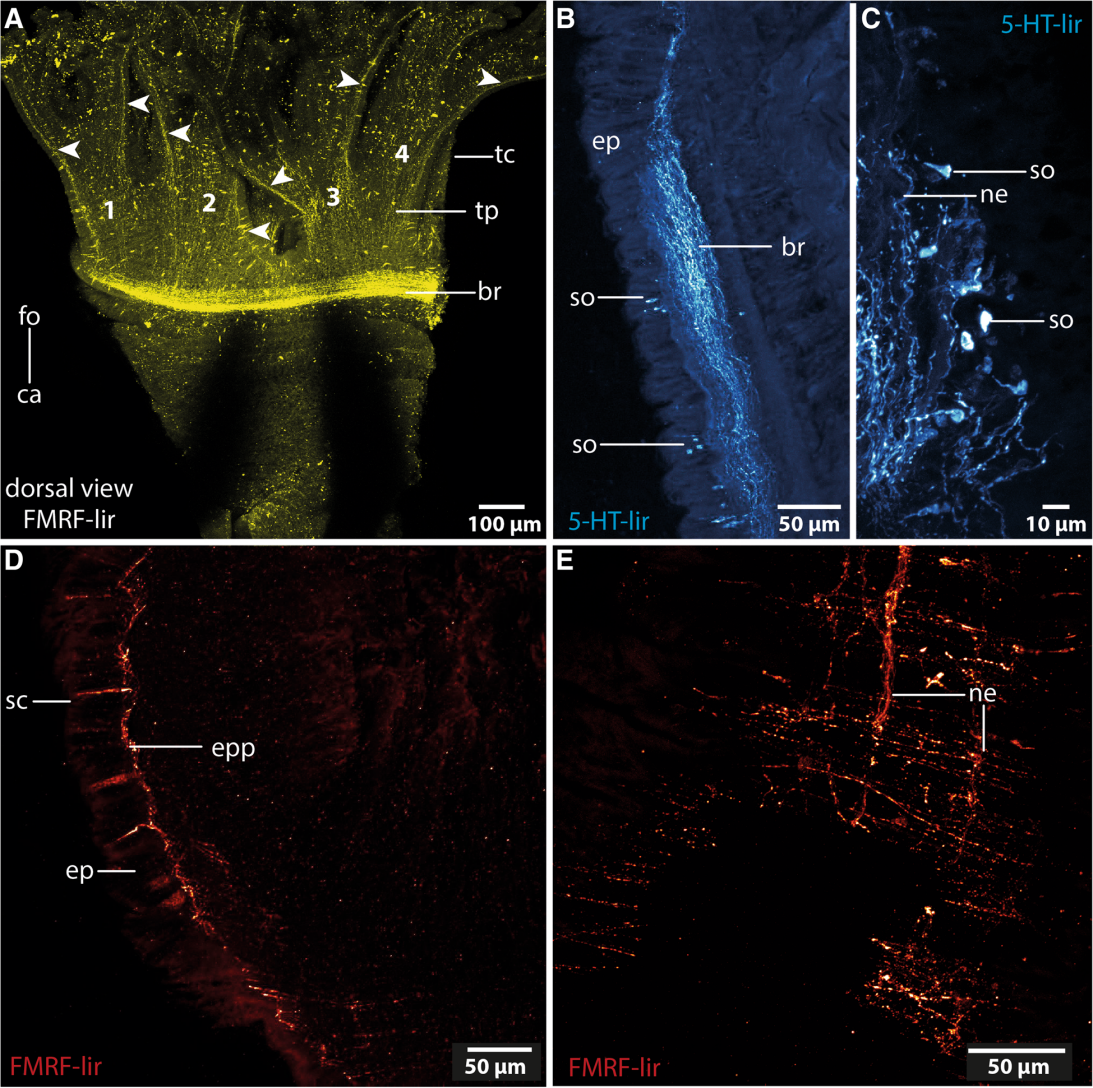
Immunohistochemistry of *Owenia fusiformis* **a**: Numbers indicate the different tentacles (1–4) of the tentacular crown. The brain ring gives rise to two main nerves (*arrowhead*) per tentacle. The tentacle epidermis contains a fine nerve plexus (*tp*). *ca*: caudal; *fo*: frontal; *tc*: tentacle crown. **b**: Lateral part of the brain ring. The neurites (*ne*) of the brain (*br*) ring are arranged in parallel. No tracts are discernable. Somata (*so*) are distributed along the course of the neuropil. **c**: Detail of B. Somata (*so*) are of the same small type. *ne*: neurites. **d**: Cross section of epidermis. Sensory cells (*sc*) are located inside the epidermis (*ep*) and are connected to the epidermal plexus (*epp*). **e**: Posterior to the brain an epidermal plexus (*epp*) is present. Neurites (*ne*) are arranged in a net-like manner

*Myriowenia* sp. possesses a pair of grooved palps attached dorsal-laterally to the head (Figs. 1a, 2a, b, 3a). Each palp contains a single large coelomic cavity lined by a simple myoepithelium. One blood vessel is present in each of the coelomic cavities (Fig. 2b). Each palp contains a basiepidermal nerve which is more prominent on the lateral quarter of palps, next the blood vessel (Fig. 2b). A giant fiber is present in each palp nerve (Figs. 2a, b). Towards the origin of the palp the nerve splits into two nerves. The more prominent one enters the brain dorso-laterally, while the smaller one enters the brain medio-dorsally (Figs. 2a, 3a). In *Owenia fusiformis* the tentacle crown consists of several branches originating from a pair of crescent lips and encompassing the terminal mouth opening (Figs. 1b, c, 3b). The branching pattern is largely dichotomous. Each of the four main branches contains a prominent coelomic space which is part of a larger pair of coelomic cavities that follow the branching pattern of the tentacles. This is also true for the paired blood vessel. The four main branches of the tentacular crown are each innervated by two main nerves which are located in the lateral parts of the flat, leaf-like tentacles (Figs. 3b, 4a). The two nerves of each branch are connected to the brain separately. Furthermore each tentacle shows a basiepidermal nerve plexus with FMRF- like immunoreactivity (Figs. 3b, 4a).

### Central nervous system

**Fig. 5:**
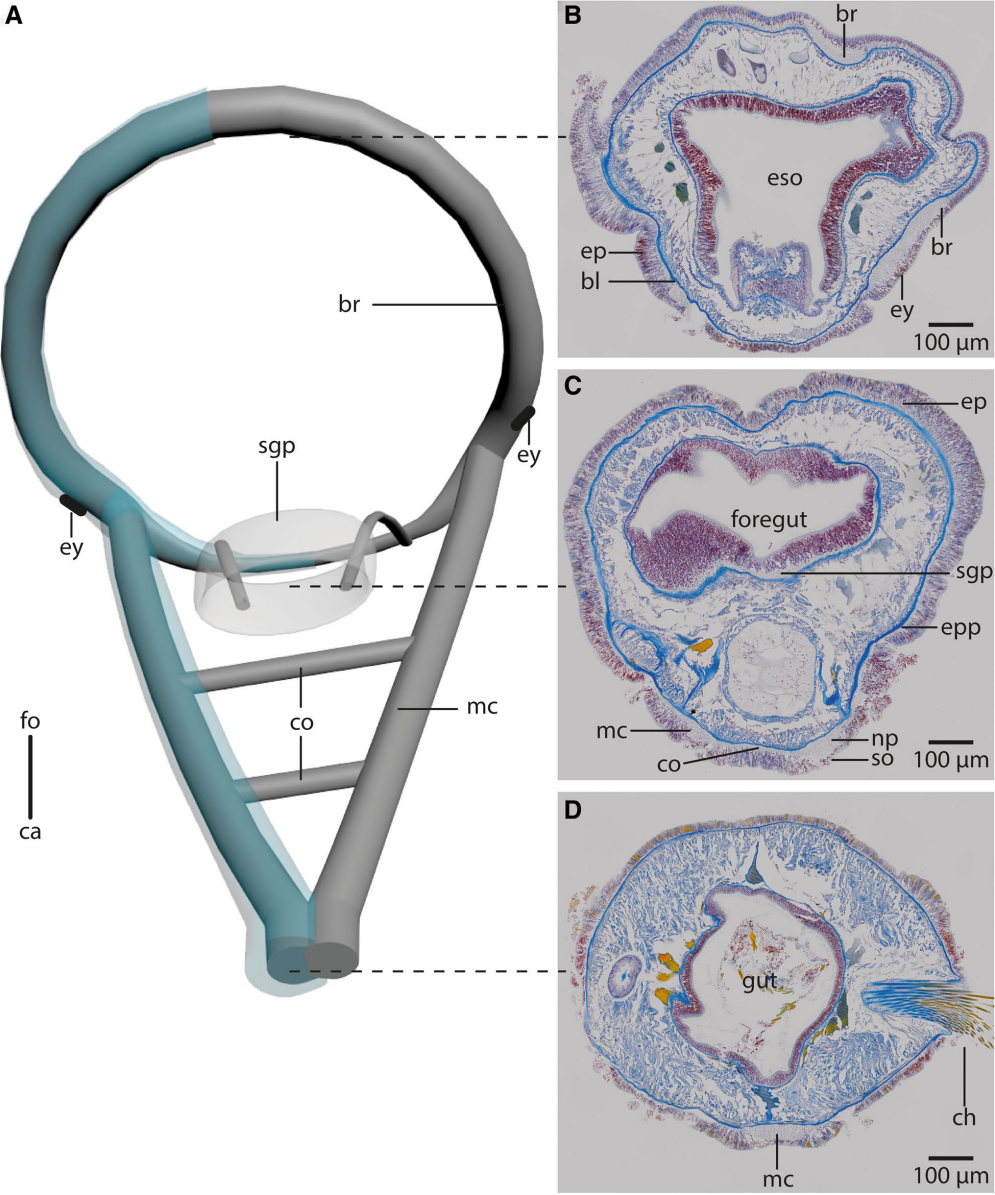
3D- model of the anterior *cns* (A), histological cross sections (5 μm), Azan staining, frontal (B) to caudal (D) of *Owenia fusiformis*. **a**: the central nervous system (*cns*, gray: neuropil, blue: somata, only drawn on the left) is composed of a ring shaped brain (*br*). Eyes (*ey*) are located ventro- laterally on the brain ring. The brain ventro- caudally gives rise to paired medullary cords (*mc*) which fuse in their further course to a single cord. The two medullary cords are occasionally connected by ventral commissures (*co*). The stomatogastric system is surrounded by a nerve plexus which arises from two nerves connected to the ventral part of the brain ring. *ca*: caudal; *fo*: frontal. **b**: the brain (*br*) is located within the epidermis (*ep*). *bl*: basal lamina; *ca*: caudal; *eso*: esophagus; *ey*: eye; *fo*: frontal. **c**: A stomatogastric plexus (*sgp*) innervates the stomatogastric system. An epidermal plexus (*epp*) is present posterior to the brain. The medullary cords (*mc*) are ventrally connected by commissures (*co*). The medullary cords are composed of a neuropil (*np*) and somata (*so*) which are located ventrally to the neuropil. **d**: Posteriorly the two cords fuse in chaetiger one to a single medio- ventrally located medullary cord (*mc*). *ch*: chaetae

**Fig. 6:**
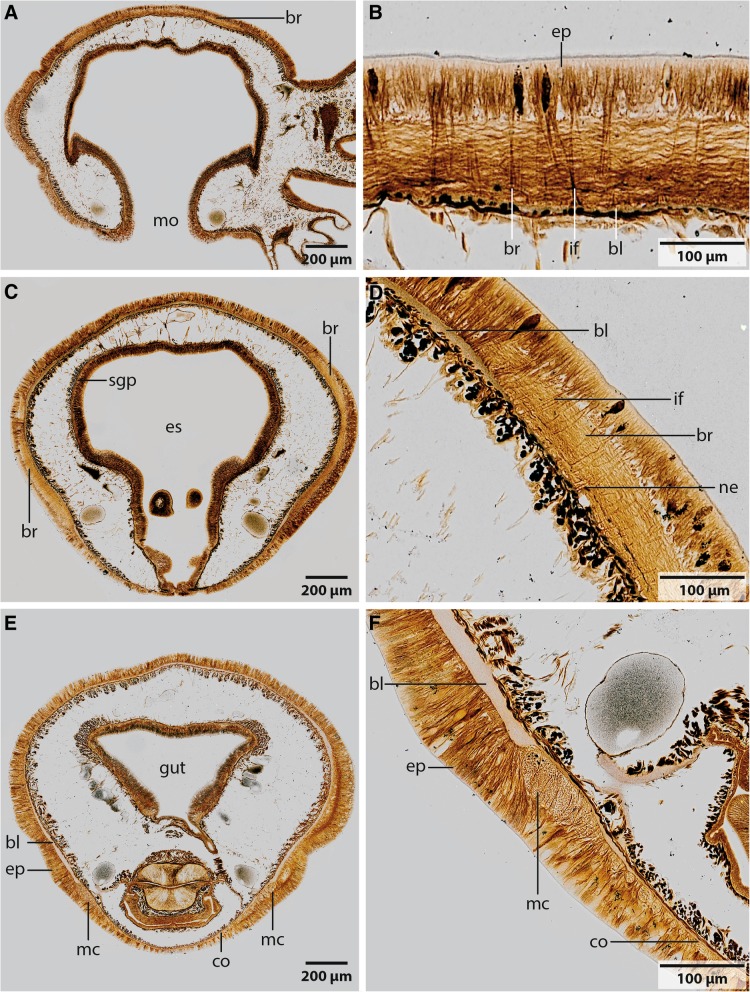
Palmgrens silver impregnation of *Owenia fusiformis*. A,C,E from frontal to caudal; B,D,F detail of picture on left. **a**: In the dorsal part of the brain (*br*) ring no commissure or tracts are discernible. *mo*: mouth. B: The brain (*br*) is located inside the epidermis (*ep*). Glia cells containing intermediate filaments (*if*) are arranged rectangular to the neurites of the brain (*br*). Cells are attached to the basal lamina (*bl*) of the epidermis. **c**: The brain (*br*) ring encircles the esophagus (*es*). **d**: The basal lamina (*bl*) is narrowed, at the points where the brain (*br*) neuropil is located. Some neurites (*ne*) are strongly stained by silver. *if*: intermediate filaments. **e**: The brain ring gives rise to paired, rectangular arranged medullary cords (*mc*). The cords are occasionally connected by commissures (*co*). The basal lamina (bl) of the epidermis is narrowed at the location of the neuropil of the medullary cords. **f**: The medullary cords (*mc*) are located inside the epidermis (*ep*), directly attached to the basal lamina (*bl*). *co*: commissure

**Fig. 7:**
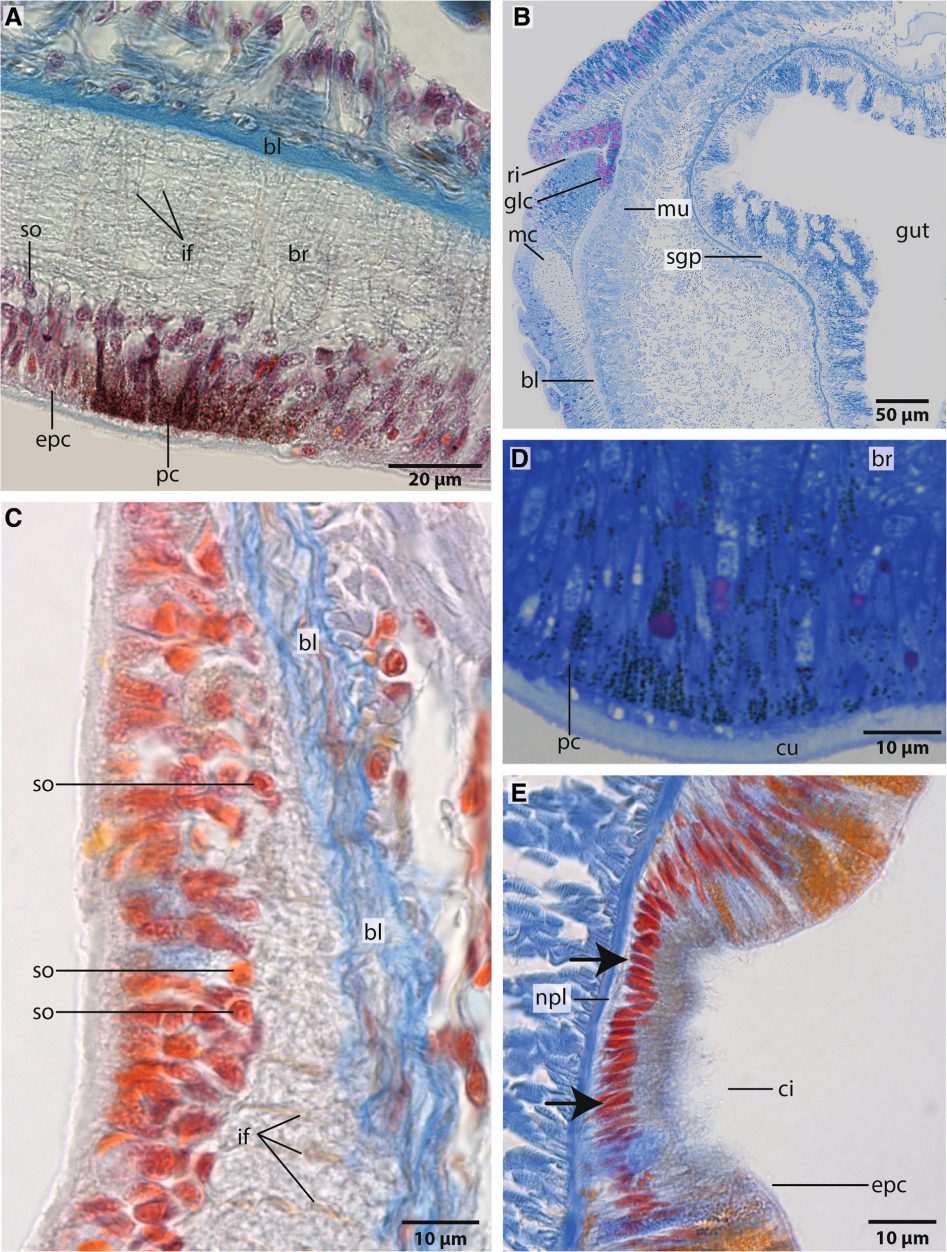
Histology, A,C,E Azan 5 μm sections, B,D: semi- thin, Toluidine-blue, 1 μm. A,B,D: *Owenia fusiformis*; C: *Myriowenia* sp.. **a**: Ventro- lateral part of brain ring. Pigmented cells (*pc*), which indicate eyes, are located between the epidermal cells. The brain (*br*) is interwoven by rectangular arranged radial glia cells (*gc*) which are attached to the basal lamina (*bl*). Somata (*so*) of the neurons are located underneath the epidermal cells (*epc*). **b**: A stomatogastric plexus (*sgp*) innervates the digestive system. Dorsal to the medullary cord (*mc*) a small rim (*ri*) is present. A cluster of purple colored gland cells (*glc*) of unknown function opens into this rim. *bl*: basal lamina of epidermis; *mu*: musculature; **c**: The brain (*br*) of *Myriowenia sp*. is interwoven by radial arranged glia cells which contain intermediate filaments (*if*). Somata of the nerve cells (*so*) are located underneath the epidermal cells (*epc*). *bl*: basal lamina. **d**: Same position as A. Pigment cells (*pc*) of the eye are arranged in a row. The brain (*br*) is located underneath the cells. *cu*: cuticle. **e**: Posterior part of specimen. In *Owenia fusiformis*, a lateral located rim is visible from the posterior part of the brain on extending posteriorly. Ciliation (*ci*) of these cells is more prominent than in the surrounding epidermal cells (*epc*). Cells (*arrows*) of the rim are lesser voluminous than in the remaining epidermis; the rim does not contain glands. A nerve plexus (*npl*) is located at the base of these cells

**Fig. 8:**
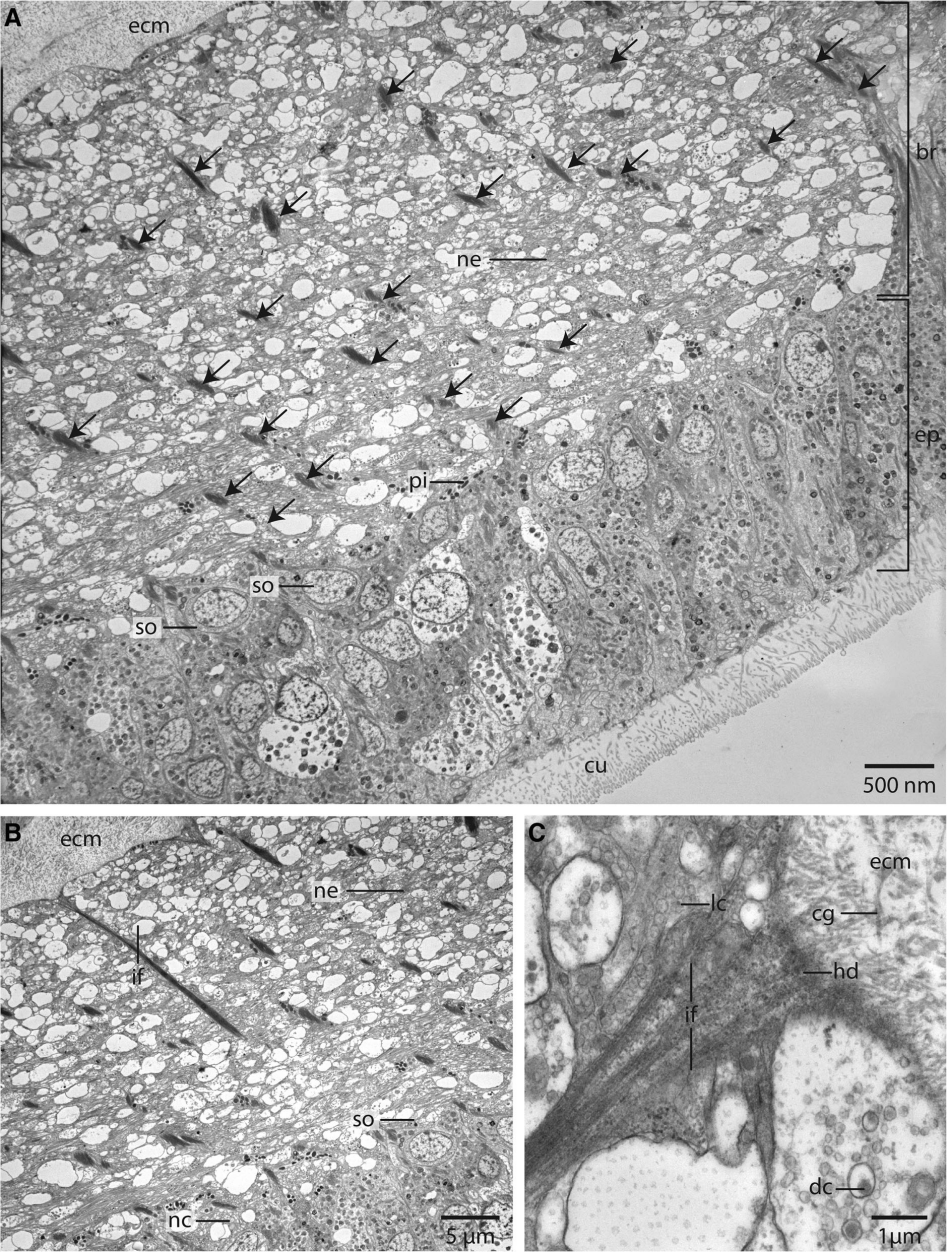
TEM, *Owenia fusiformis*. **a**: The neurites (*ne*) of the neuropil of the brain (*br*) are attached to the basal lamina (*ecm*) of the epidermis (*ep*). Somata (*so*) of the nerve cells are intermingled between the epidermal cells. The neuropil is interwoven with radial arranged glia cells, which contain dense bundles of intermediate filaments (*arrows*). *cu*: cuticle. **b**: The intermediate filaments (*if*) are attached to the basal lamina of the epidermis. Neurites (*ne*) are of different size. *nc*: nucleus; *so*: somata of nerve cells. **c**: Neurites contain lucent core (*lc*) as well as dense core (*dc*) vesicles. Intermediate filaments are attached to the *ecm* via hemidesmosoms (*hd*). Collagen fibres (*cg*) are well visible in the basal lamina (*ecm*) of the epidermis

In both species the anterior central nervous system (*cns*) consists of a ring-shaped brain surrounding the mouth opening and a ventral medullary cord (Figs. 1c, 2a, 5a). The *cns* is composed of a central fibre core (neuropil) and surrounding somata of the neurons. The fiber core consists of neurites; its medial face rests on the basal lamina, thus occupying a basiepidermal position (Figs. 2; 5, 6, 7a, c, 8). Somata surround the lateral and outward aspects of the neuropil, but are missing where it rests on the basal lamina (Figs. 2, 5, 6, 7a-c, 8a). Monociliated epidermal cells surround and overarch the neuropil, so that their nuclei and those of the neurites can hardly be discriminated in histological sections. Neuron specific antibodies against FMRF-amide and 5HT, however, show that in *Owenia fusiformis* the somata are located between the epidermal nuclei (Figs. 4b, c). The neurons are of the same small type (type 1 neurons, which is defined by the little cytoplasm around the nucleus) (Figs. 7a, c). Due to the basiepidermal position of the central nervous system, an outer and inner neurilemma separating somata of the neurons from the neuropil are missing (Figs. 2, 5, 6, 7). Radial glia cells cross the neuropil (Figs. 6b, 7,a c, 8). In both species somata are evenly distributed along the whole course of the central nervous system and ganglia are absent (Figs. 2, 4, 5, 6).

The brain is oriented slightly oblique to the anterior-posterior body axis, so that its dorsalmost part lies posterior to the mouth opening and its ventralmost section anterior to the mouth. In *Myriowenia* sp. the nerves innervating the palp originate from the dorso-lateral aspects of the brain ring (Figs. 2, 3a). The dorsalmost part of the brain ring continues between both palp nerves; thus its fiber core is smaller in this area but still surrounded by somata (Fig. 2a, c). A pair of stomatogastric nerves arises from the lateral section of the dorsal part of the brain ring in *Myriowenia* sp. (Fig. 2a). These nerves soon ramify and form the stomatogastric plexus (Fig. 2d). The ventral nerve cord originates from the ventralmost section of the brain (Fig. 2a; e). Except for the dorsalmost section, the pronounced fiber core bulges the epidermis, so that the course of the brain ring can roughly be seen from exterior (Figs. 1a, 2d). In *O. fusiformis* the ventral nerve cord arises from two perpendicular arranged medullary cords that branch off ventro-laterally on either side of the brain ring on the level of the mouth opening (Fig. 5a). The lateral located medullary cords are interconnected by two non-segmental commissures prior to them merging into a single medullary cord from the first chaetiger onwards (Figs. 5a, d). These commissures are not discernable in μCT- scans (Fig. 1c-e). The ventralmost section of the brain ring is located between the two rectangular medullary cords. The neuropil is smaller in this section, but still surrounded by somata (Fig. 5a, c). A pair of stomatogastric nerves branches off the ventral brain section. They soon ramify forming a plexus which innervates the foregut (Figs. 5a, c, 7b). In contrast to *Myriowenia sp.* the fiber core of the brain does not cause epidermal bulging, since the diameter of the basal lamina is reduced where it underlies the brain and medullary cord (Figs. 6d, f, 7b). In *O. fusiformis* an epidermal nerve plexus is present (Fig. 5c). The plexus gives rise to neurites which presumably belong to sensory cells inside the epidermis (Fig. 4d). Neurites of the epidermal plexus form a network (Fig. 4e).

The giant fibre that runs inside each palp nerve cord in *Myriowenia* sp. follows the course of the brain ring. At the origin of the ventral nerve cord they fuse into a single median giant fiber, located in the dorsal portion of the neuropil (Fig. 2). After fusion, the giant fibre is considerably larger in diameter (appr. 34 μm) than both single fibres (appr. 12 μm each) together. Such giant fibres were not found in *Owenia fusiformis*.

### Ultrastructure

**Fig. 9:**
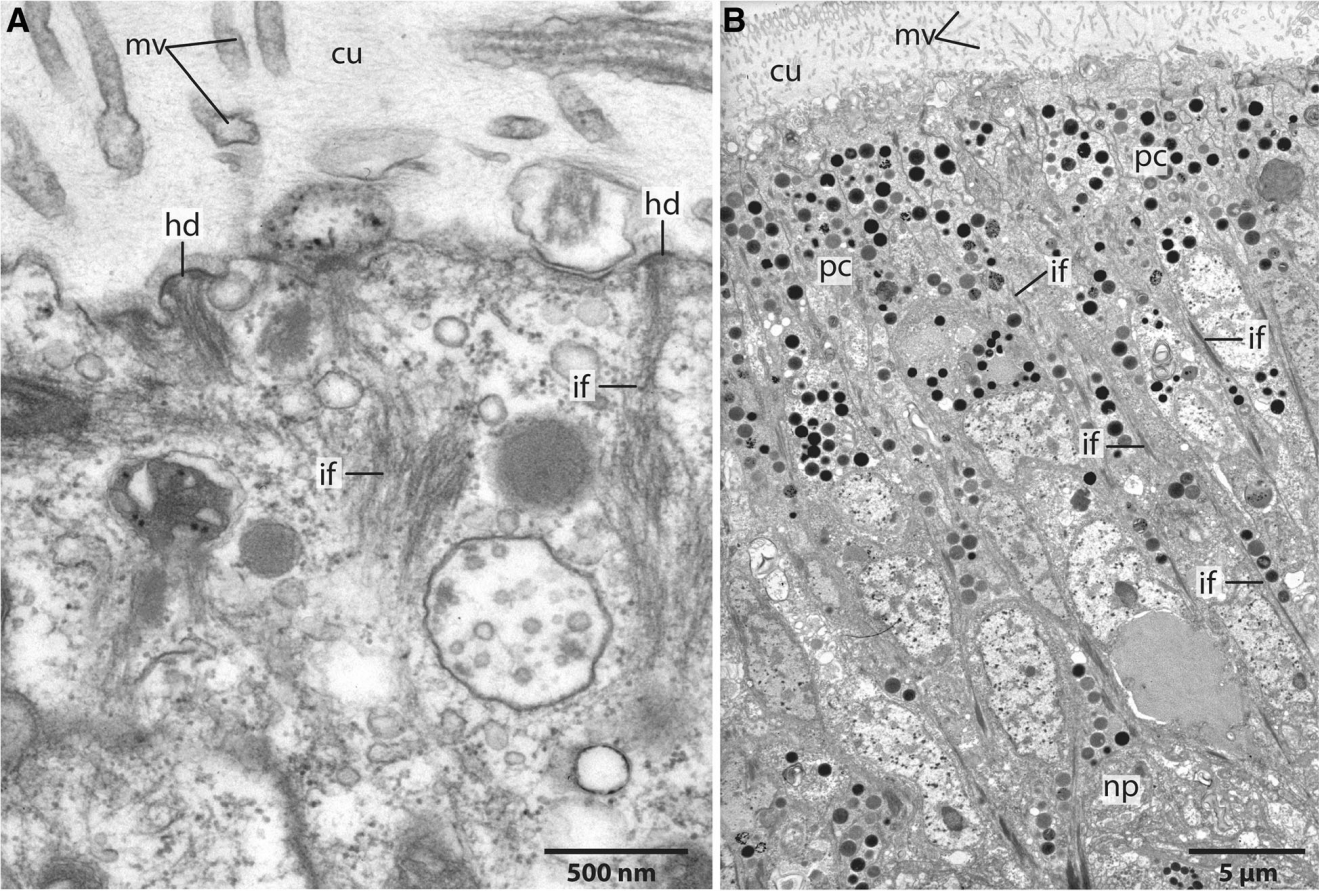
TEM, *Owenia fusiformis*. **a**: The intermediate filaments (*if*) are attached to the cell surface by hemidesmosoms (*hd*). Microvilli proceed (*mv*) into the cuticle (*cu*). **b**: eye spot shown in Fig. 1a. Pigment cells (*pc*) are arranged in a row. The apical parts of the cells possess microvilli (*mv*) which extend into the cuticle (*cu*). The brain neuropil (*np*) is located underneath the eye. *if*: intermediate filaments

The neuropil of the brain of *Owenia fusiformis* and *Myriowenia* sp. is interwoven by a dense network of rectangular arranged glia or radial glial cells (Figs. 6b, c, 7a, c, 8). These glial cells possess a broad apical section and a slender basal process adhering to the basal lamina. These monociliated cells are an integrative part of the epidermal cell layer and typical cell junctions connect them to their neighbors (Fig. 9a). Their most conspicuous feature is a basally-apically oriented system of bundles of intermediate filaments. Occasionally the intermediate filaments ramify basally (Fig. 8c). The intermediate filaments are densely packed inside the slender processes of the glia cells and cause their characteristic histological and ultrastructural appearance (Figs. 6b, d, 7a, c, 8). In Azan staining the intermediate filaments inside the radial glial cells appear as twisted, yellow bundles (Fig. 7c). Apically, hemidesmosomes connect the intermediate filaments to the cuticle and basally to the lamina densa of the basal lamina (Figs. 8b, c, 9a). Neuronal somata are distributed along the neuropil and partly intermingle with the cell bodies of the epidermal cells which are located more or less above the neuronal somata. These are not enwrapped by glial cell processes (Fig. 8a). Neurites of the brain are arranged in parallel and are of different diameter (Figs. 4a, b, 8a, b).

The size of the nerve cell somata is fairly uniform. The neurites and often also the somata possess numerous vesicles that differ in stainability of their content. We recorded dense core vesicles indicating neuropeptides as well as vesicles with homogenous filling of differing electron-density ranging from clear to electron-black fillings in different neurites (Fig. 8c). Beside the vesicles we also found small mitochondria ranging between 220 and 270 nm in diameter as indicative for nerve cells. Certain cells contain intermediate filaments and larger ovoid and electron-dense vesicles. Both are indicative for glial cells; the vesicles are assumed to be gliosomes.

### Sensory organs

*Owenia fusiformis* possess a pair of ventrolateral, crescent structures that differ in pigmentation from the surrounding epidermis. These structures are regarded as eye spots (Figs. 1a, 7a). Azan and toluidine stained sections show a unique pigmentation of cells arranged in a flat patch (Figs. 7a, d, 9b). A cup-like structure is absent. The presumed eyes are located next to the ventro-lateral section of the brain ring. This region consists of slender cells containing numerous electron-dense vesicles measuring 480 ± 20 nm (*n* = 10). The pigment forms an almost complete apical layer in this region; merely a few unpigmented cells pierce this layer (Fig. 9b). Most of the pigmented cells contain intermediate filaments that cross the cell in a baso-apical direction (Fig. 9b). Their basal section is slender; here, the intermediate filaments form dense bundles that are visible as electron-dense rods that cross the basiepidermal nerve plexus.

No such differently pigmented structures were found in *Myriowenia* sp.

**Fig. 10:**
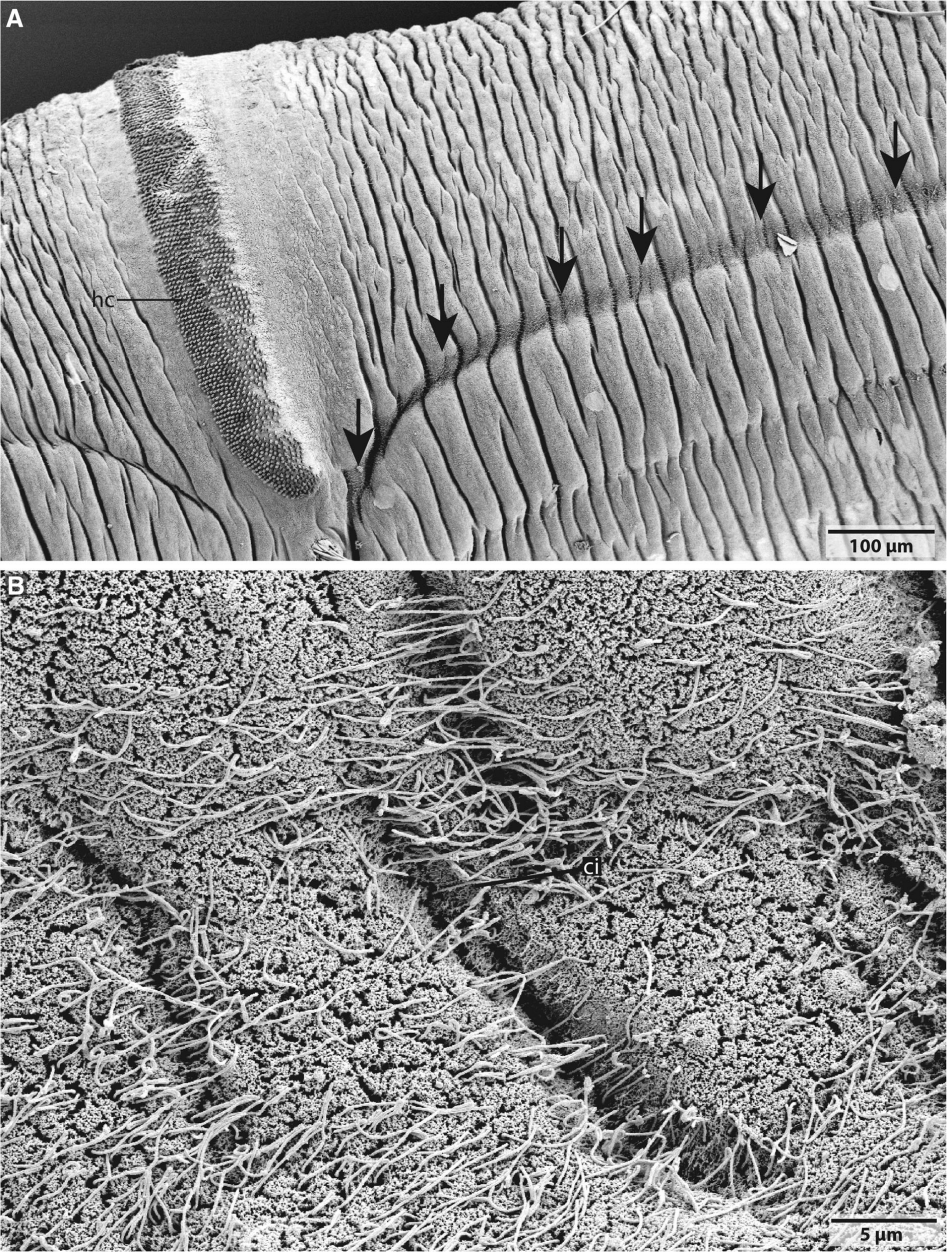
SEM, *Owenia fusiformis.* **a**: The lateral located rim (*arrows*) terminates close to the hooked chaeta (*hc*) field and proceeds anteriorly. **b**: The rim is densely ciliated (*ci*)

In *Owenia fusiformis* a continuous lateral rim is visible on either side of the body (Figs. 7b, c, 10a, b). This rim is densely ciliated and is underlain by a plexus that projects into the lateral or ventral nerve cord (Figs. 7b, e, 10). The cells that form this rim are slender and possess a small apical area. Some of them are biciliated. All cells contain a specific pigmentation, no neurites connecting these cells to the nerve plexus were found. No ciliated rim or any ciliated areas between neuropodial and notopodial chaeta were found in *Myriowenia* sp.

Uniciliated penetrative cells or collar receptors were not observed in *Owenia fusiformis.* Likewise a nuchal organ is absent from both species.

## Discussion

### Comparison of present and previous studies of Oweniidae nervous systems

Recent phylogenomic studies of Annelida univocally show Oweniidae and Magelonidae (most likely together in the common clade Palaeoannelida) to branch off as sister group to the remaining groups of Annelida [11, 18].

Besides our studies of *Myriowenia* sp. and *Owenia fusiformis* we had the opportunity to look into unpublished sections of the nervous system in *Owenia fusiformis* and *Magelona papillicornis* done by Orrhage. We are aware that Orrhage’s *Owenia* species may not correspond to our *O. fusiformis* species [40]. Despite this and the fact that his sections were stained with a different dye, they show the same details and results as our histological sections of *O. fusiformis*. This close correspondence justify a broader comparison of our results to the brain schemes set up by Orrhage on e.g. Magelonidae [36].

Phylogenetic relationships among Oweniidae have long been a matter of debate [41, 42]. The most recent study on oweniid in-group relationships [43] showed *Owenia fusiformis* and *Myriowenia sp*. to be nested in each of their different clades in the tree, making them good representatives for inferring the ancestral structure of the oweniid central nervous system and sense organs. In both species the central nervous system is basiepidermal, a position which is also known for other closely related Annelida like Magelonidae and Chaetopterifomia as well as for numerous pleistoannelid taxa [2, 11]. Thus, a basiepidermal central nervous system is proposed as representing not only the primary condition for Oweniidae but also as the ancestral state for annelids.

In both species studied the brain is a simple, basiepidermal ring that is continuous with the ventral nerve cord and shows no anterior split into a dorsal and ventral root of each lateral cord in adults. In *Owenia fusiformis* the ventral nerve cord arises from two perpendicular arranged lateral medullary cords, in contrast to *Myriowenia* sp. where the ventral nerve cord directly arises from the midventral part of the brain. Since Magelonidae also possess a pair of lateral medullary cords, arranged perpendicular to the brain [36], it is likely that two initially separated ventral medullary cords with non-segmental commissures, like in *Owenia fusiformis*, represent the primary condition. Therefore, a single ventral cord in *Myriowenia* must be a derived character state. Müller [28] stated that in the ground pattern of annelids the circumesophageal connectives have two roots. However, in presumably basally branching groups within annelids, such as Magelonidae, Apistobranchidae and Psammodrilidae circumesophageal connectives do not exist, since the whole *cns* is medullary (own unpublished observation). Moreover, the brain is ring-shaped, surrounds the terminal mouth and gives rise to the ventral cord without separate neurite bundles. This statement differs significantly from Orrhage’s extensive studies of the annelid nervous system in other groups ([1] for review). As mentioned above we also studied Orrhage’s original sections of *Magelona papillicornis* and were not able to reach the same conclusions as Orrhage [36], but this issue will be part of a follow- up investigation.

Regarding the presence of head appendages, all species of *Myriowenia* possess one pair of palps [43]. Although unique among Oweniidae, they must be regarded as a synapomorphy of Palaeoannelida and the remaining annelids, since these paired feeding appendages which are found in most of the closely related annelid lineages, the so-called grooved palps, are assumed to be homologous across Annelida [1, 14]. However, a common origin of palps within Annelida has been debated since the first anatomical investigations on the annelid nervous systems were performed in the middle of the nineteenth century. Orrhage and Müller [1] give a comprehensive review on this topic (see also Gustafson p. 372 for historical background [44]). In both species studied here, two pairs of nerves branch off from the brain and enter either the palps of *Myriowenia* or enter into each half of the tentacle crown in *Owenia fusiformis*. These nerves do not only indicate homology of the tentacle crown of *Owenia* with the palps of *Myriowenia*, they also suggest two pairs of palp nerves in the oweniid stem species. Since magelonids and chaetopterids also possess a pair of palps with a similar innervation pattern [36], such types of palps are most likely the primary condition in Oweniidae and Palaeoannelida. Within Oweniidae they were either modified into a tentacle crown or were completely reduced in certain oweniid species such as *Galathowenia* and *Myrochele*. Therefore, in all probability this pattern must have been present in the last common ancestor of annelids as well and all other patterns observed represent derived character states.

A giant fibre is present in *Myriowenia* sp., but absent in *Owenia fusiformis*. Since magelonids and various other annelids [3] possess such fibres, their homology and secondary reduction in *Owenia fusiformis* seems to be the most parsimonious explanation.

Pigmented eyes are missing in *Myriowenia* sp., but are present in *Myriochele* and *Owenia fusiformis* and described for *Galathowenia* species [34, 43]. The structure of these eye spots differs from that present in other annelids e.g. [2, 12, 45]. Usually annelid eyes are cup-shaped and comprise two cell types: rhabdomeric photoreceptor cells and pigment cells with shading pigment. The phylogenetic significance of the eyes in Oweniidae is presently hard to evaluate. A detailed comparative study on the eye structure in oweniids and other annelid taxa is underway to clarify this question and to better understand the evolution of eyes in annelids.

Until now nuchal organs are regarded as one of the most important apomorphies of Annelida [14, 15]. Consequently, their absence in annelids has always been regarded as a loss and thus as secondary [12 for review]. However, in the light of the current molecular phylogenies this hypothesis must be reconsidered. Since our re-investigation verifies the absence of nuchal organs in adult Oweniidae and previous examinations support the lack of these structures in Magelonidae as well as in Chaetopteriformia [46], we hypothesize that this conspicuous sensory organ must have evolved within Annelida, in the stem lineage of the clade comprising Amphinomidae/Sipuncula and Pleistoannelida (Errantia + Sedentaria). Consequently, nuchal organs are not considered an apomorphic trait of Annelida (see also [2]).

Another type of prominent sensory organs - the lateral organs described for magelonids and other annelids are absent in Oweniidae. The laterally located, densely ciliated groove in *Owenia fusiformis,* which was described as a potential sensory structure by McIntosh [39], most likely has no sensory function since no connection of the ciliated cells to the nervous system was found in the present study.

Presently, there is no evidence that complex sensory organs such as elaborated eyes, nuchal organs or lateral organs were present in adults of the last common ancestor of annelids.

### Simplicity in Oweniidae is not necessarily secondary

One could argue that the low complexity of the central nervous system in oweniid species and the lack of complex sensory organs results from the tube-dwelling mode of life and therefore, is secondary. If so, one would expect that the nervous system is also simple in those sedentary groups that are more derived within the annelid tree, like Sabellariidae or Sabellidae. Both comprise tube-dwelling species which should then accordingly have a comparable and structurally similar, “simple” nervous system. However, this is not the case. Although, the nervous system of e.g., *Sabellaria alveolata* shows less complexity compared to those of errant families such as Eunicidae, it still comprises a ganglionic, rope-ladder like ventral nervous system, a comparatively complex subepidermal brain and complex sensory structures such as nuchal organs [2, 11, 47, 48].

However, taking developmental studies into account the picture seems more complex. The brain of *Owenia fusiformis* initially shows several commissures in staining against anti- 5HT which fuse during development ([33], Fig. 4h). The same is true for *Magelona filiformis* (Helm, unpublished); however, these brain commissures are not retained as separate commissures in the adult stage. The annelid ancestor may likewise have had additional commissures during development, but there is no support yet for their presence in the ancestral adult stage. Moreover, it is unknown whether commissures in errant annelids like eunicids are incorporated from the larval stage into the adult brain or if these commissures develop de novo, emphasizing the need for more detailed studies into nervous system development to clarify these issues.

Given Orrhage’s reconstruction of the anterior nervous system in *Magelona filiformis* [36] was correct and we were simply not able to see his observation properly (unpublished study, not shown here), the presumed sister group of Oweniidae would have two roots of the circumesophageal connectives. In this case these structures may have been secondarily reduced in Oweniidae, except if Magelonidae turns out to be the sister group to the remaining Annelida (minus Oweniidae). This unresolved situation shows the necessity for further studies into the annelid nervous system on the one hand and a more thorough look into the potential outgroups on the other. In this respect a special focus should also be laid on the diversity of nerve cell types, as Magelonidae possess a higher diversity of nerve cell types than Oweniidae.

### Comparison within Annelida and next to spiralian outgroups

For discussion of the evolution of the nervous system of Annelids we use the phylogeny presented by Helm et al. [11]. Since it still is a matter of debate which lophotrochozoan taxon actually constitutes the sister group of annelids, we discuss characters of several of the putative next relatives among Spiralia, such as Nemertea, Mollusca, Phoronida, Bryozoa, and Brachiopoda [19, 49, 50].

*Position of the cns.* A basiepidermal position of the nervous system such as present in oweniid species is also found in certain but not all taxa of Sedentaria (e.g. Cirratulidae) and Errantia (e.g. *Nephtys hombergii*) [2, 3]. A subepidermal position of the nervous system is found in taxa of the Eunicida (e.g. *Marphysa bellii*) and in some taxa of the Sedentaria (e.g. *Sabellaria alveolata*) [3, 11]. In species representing sister taxa to remaining taxa within related lophotrochozoan lineages such aslike Nemertea [51], Brachiopoda and Phoronida the central nervous system is also basiepidermal [3, 52, 53]. In Mollusca the nervous system is exclusively subepidermal (intramuscular) [3, 54–57]. Using the most recent phylogenies of Spiralia as backbone [49, 50], the basiepidermal position of the *cns* most likely represents the plesiomorphic condition in Spiralia and indicates that the shift of the nervous system beneath the basal lamina of the epidermis into the mesodermal tissue (musculature) occurred secondarily and repeatedly within the different spiralian taxa.

*Brain.* In the Oweniidae species investigated the brain is circular and surrounds the mouth opening. There are no dorsal enlargements, lobes or commissures/tracts. Neither a supraesophageal nor a subesophageal ganglion (or any dorsal clusters of somata) is present; ganglia were also not found in the ventral nerve cord. The homogenous distribution of somata forming a crescent layer around the neuropil classifies the entire central nervous system as medullary and not ganglionic [5]. Posterior to the mouth opening the neuropil of both body sides fuse to form a single cord. This morphology is the simplest with respect to somata distribution and the enlargement of the dorsal region of the brain found in Annelida (see also [2, 34, 38]).

In other spiralian taxa like phoronids the *cns* is simple, consisting of a dorsally located brain with no enlargements and an epidermal nerve plexus in the remaining body. Ganglia and polymorphic neuron clusters are not reported [49]. In Brachiopoda the brain is also simple with respect to somata distribution and dorsal enlargements [53]. In Broyozoan the *cns* is a neuroepithelium and polymorphic neurons are reported. [58, 59]. The most basally branching taxon of Nemerteans also possess a circular brain, a medullary *cns* and only slightly dorsal enlargements of the brain [51, 60]. In mollusc taxa Caudofoveata, Solenogastres and Polyplacophora the *cns* is also medullary, the brain is more or less circular with no dorsal enlargements and gives rise to two lateral (visceral) and two ventral (pedal) medullary cords [54, 61]. All these findings indicate that a simple medullary, ring-shaped brain represents the ancestral condition in annelids, and most likely also a plesiomorphy of Annelida as well as several other spiralian lineages.

Within Annelida the dorsal aspect of the brain is enlarged by evolving ganglia, additional fiber cores in the brain and clusters of polymorphic neurons. This also applies for other taxa, like Nemertea and Mollusca [3, 60, 62]. Along with such an expansion of the brain, higher brain centers such as the mushroom bodies, evolved convergently within Annelida and presumably other metazoan groups [63]. Evolution of brain complexity within different spiralian lineages might explain traditional difficulties to homologize certain brain areas across Spiralia [63]. Based on the recent phylogenies higher brain centers most likely evolved independently in different taxa, so that any attempt homologizing them is futile.

In Oweniidae only one type of neurons prevails in the central nervous system. In contrast to other neurons this small type of neuron possesses a very small cell body around the nucleus. [3]. Additional neuron types evolved in the stem lineage of Pleistoannelida+ Sipuncula/Amphinomidae [2, own unpublished observation]. In basally branching lineages within Nemertea [51] and Polyplacophora ([51] for review,) only one class of neurons is also present, while the number of different polymorphic neuron classes increases independently within all larger spiralian groups, including Annelida [3, 60, 62].

*Glia.* Conspicuous are the intermediate filaments which run through the neuropil of the *cns* of both species investigated*.* These filaments form bundles in glial cells and are attached to the basal lamina of the epidermis via hemidesmosoms. These glial cells are called fibrous glia [64] or radial glial cells [65]. According to their position and ultrastructural details radial glial cells represent modified epidermal cells. In contrast to the latter, they are able to secrete a protein called SCO-spondin which contributes to the formation of Reissner’s fibre [65]. Glia cells are distinguishable from epidermal cells, since they possess gliosomes and dense bundles of intermediate filaments. In epidermal cells intermediate filaments are also present and attach these cells to the basal lamina, but do not form such prominent bundles. Intermediate filament bundles are thick, twisted yellow bundles in Azan stained histological sections. While they are clearly visible in the nervous system they are not present in the epidermis. The same kind of cells are also found in Phoronida [66], Nemertea [67] and Polyplacophora (own unpubl. observation) indicating an early evolution of these cells. Moreover, in errant annelids glial cells form a cortex around the neuropil layer [2, 68, 69]. Baskin regarded this massive layer of glial cells to be important to prevent mechanical stress to the nervous system while moving [68, 69]. In so far investigated species representing sister clades to remaining clades within the major Spiralian taxa, a glial- cell layer surrounding the brain is not present. Radial glial cells with a prominent intermediate filament system seem to be especially important in species which possess a basiepidermal nervous system, since the epidermal cells which overlie the neuropil have to be attached to the basal lamina. Additionally, this basiepidermal fiber system is important to maintain the structure of the epidermis and the underlying *cns*.

#### Sense organs

As outlined above, the so-called eye spots in oweniids are presently difficult to evaluate due to their absence in some oweniid species and missing information on the eye structure in other basally branching annelid taxa. The eyes of *Owenia fusiformis* do not correspond currently to any type of eye found in the remaining annelids and are missing in the other oweniid taxon investigated, *Myriowenia*. Structurally these eyes somewhat resemble those of larval polyplacophorans or some gastropods such as limpets in consisting of a simple monolayer of epidermal pigment- and sensory cells [70].

## Conclusion

Our data support a basiepidermal position of the *cns* in the annelid stem lineage since this is the position found in the families which are close to the base of the tree like Oweniidae, Mageloniidae, Chaetopteriidae, Apisthobranchidae and Psammodrilidae [2, 11]. In Oweniidae the brain is ring-shaped and confluent with two perpendicular arranged medullary cords which fuse posterior to a medio-ventrally located single medullary cord. There is no evidence for the anterior medullary cords each splitting up into a dorsal and ventral root lateral to the brain in the investigated species of Oweniidae, but this split has been found in other basally branching lineages [36]. Nonetheless, these antero-lateral parts of the cords in Oweniidae cannot be termed circumesophageal connectives because the entire *cns* is medullary and thus they represent nothing else than a part of the brain. Moreover, there is no distinct border between brain and ventral nervous system. Neither ganglia nor mushroom bodies nor glomerular neuropil bodies are exhibited within the brain of Oweniidae. Since these structures are also absent in outgroup taxa and only present in Errantia [8], they were putatively also not present in the stem lineage of Annelida. On the other hand, grooved palps were most likely present in the last common ancestor of Annelida, and each palp was innervated by two nerves originating from the dorsal part of the brain. Nuchal organs were not present as well and thus must have evolved in the stem lineage leading to the clade containing Amphinomida, Sipuncula and Pleistoannelida. Similarly, presence of lateral organs as sensory structures between neuro- and notopodium cannot be parsimoniously traced to represent an ancestral trait. Although a pair of adult eyes most likely was present in the ancestor of annelids, their homology across annelids cannot be assessed due to the lack of detailed information regarding opsins and development as well as structure, especially in the non-pleistoannelid taxa.

## Material and methods

### Specimens

Specimens of *Myriowenia sp.* Capa, Parapar, Hutchings, 2012 were collected in New South Wales, Australia (Fig. 1a). *Owenia fusiformis* Delle Chiaje, 1844 (Fig. 1b) were found in the sandy flat of Pouldohan (Tregunc, Finistere), close to the city of Concarneau (Brittany, France) in August 2016.

The original sections that Lars Orrhage used for his reconstructions of the annelid nervous system are deposited at the Stockholm Naturhistoriska riksmuseet curated by Lena Gustavsson. We analysed and photographed Orrhage’s unpublished sections of *Owenia fusiformis* to evaluate our results and conclusions. His sections were stained with hematoxylin and eosin. Hematoxylin gives fibres (intermediate and actin filaments) and nuclei a dark staining. Sections thickness is 5 μm (Additional file 1: Figure S1).

### Parafin- histology

Animals were relaxed in a 7% MgCl_2_ solution mixed with seawater 1:1 and then fixed in Bouin’s fluid (stock solution: 1 g Picrid acid solved in 100 ml Ethanol), (80 ml stock solution + 20 ml 35% Formol + 2 ml of Acetic acid) for 12 h, dehydrated in an ethanol series, followed by ethanol-butanol and butanol and preincubated in Histoplast (Thermo Scientific, Dreieich, Germany) at 60 °C. After three days with several medium changes they were finally embedded in Paraplast (McCormick Scientific, Richmond, USA). Serial sections of 5 μm thickness were performed using a microtome (Autocut 2050, Reichert-Jung, Leica, Wetzlar) and transferred to glass slides coated with albumen-glycerin. Two specimens of *Myriowenia sp*. and *Owenia fusiformis*, respectively were stained with Azan. One specimen of *O. fusiformis* was stained with Palmgrens silver impregnation.

### Palmgrens silver impregnation

Sections were transferred to Xylol to elutriate Paraplast and were transferred from a Xylol-Ethanol (1:1) solution into a descending ethanol series (100, 95, 90, 80, 70% + nine drops of ammoniac to intensify the following reduction process, 40% ethanol). The sections were washed in distilled water and transferred in a Formol-nitric-acid-distilled water solution. The sections were again washed in distilled water and then stained in a silver nitrate solution before they were transferred to a 40 °C tempered reduction solution. Then, the sections were transferred via 50% ethanol and distilled water in a 5% sodium thiosulphate solution. Subsequently, the sections were washed in tap water and were transferred via an ascending ethanol series (40, 70, 90, 95, 100% ethanol) to Xylol.

### Immunohistochemistry

Six specimen of *Owenia fusiformis* were relaxed for immuno-histochemical investigations in a 7% MgCl_2_ seawater (1:1) solution and subsequently fixed in 4% paraformaldehyde in seawater. Animals were embedded into Gelatine-albumen (Sigma- Aldrich) and cut into sections of 60 μm thickness with a vibratome (HM 650 V Thermo-Scientific) or intact animals were used as whole mounts. Animals were stained with antibodies against FMRF-amide (ImmunoStar, Hudson, WI, USA), acetylated α-Tubulin (Sigma-Aldrich, Saint Louis, MO, USA) and for the nucleus staining Sytox (Invitrogen, Carlsbad, CA, USA). For a detailed description see Beckers et al. [51]. For whole mount staining the tissue of *Owenia fusiformis* was permeabilized using 2% Triton X-100 in PBS for 65 h in the fridge. Antibodies of rabbit anti- FMRF-amide (ImmunoStar, Hudson, WI, USA) and mouse α-tubulin (Sigma-Aldrich, Saint Louis, MO, USA) were applied for 3 days after blocking in 6% swine serum in PBS containing 0.5% Triton-X-100. Secondary antibodies were applied for 2 days at a dilution of 1: 1000. Animals were treated with Murray clear (benzyl benzoate + benzyl alcohol) and embedded therein on glass slides. Vibratome sections were embedded using Elvanol on glass slides. Afterwards the slide preparations were scanned with a Leica TCS SPE CLSM. Image stacks were further processed using Fiji (1. 52 h).

### μCT

Specimen of *Owenia fusiformis* used for μCT investigations were fixed overnight using Bouin’s fluid. Animals were washed in 70% Ethanol and stained with 0.3% PTA (Phosphotungstenacid) in 70% Ethanol for 1 week. Afterwards animals were scanned with a μCT (Skyscan 1272, Burker, Germany) at 600 nm resolution. Image stacks were further processed using Fiji (1. 52 h).

### SEM

Specimen of *Owenia fusiformis* used for SEM investigations were fixed using Bouin’s fluid, dehydrated and critical point dried (Bal-Tec CPD 030, Switzerland). Afterwards, the specimens were sputter coated with gold (SEM coating unit E5100, Polaron Equipment Ltd., Great-Britain), mounted on aluminum stubs and studied in a Philips XL30 ESEM.

### Semi- thin sectioning and TEM

Anterior parts of *Owenia fusiformis* were fixed in a 2.5% glutaraldehyde solution buffered in 0.05 M phosphate buffer 0.3 M saline (pH 7.2) at 4 °C for 2 h and kept in the same buffer. The specimens were postfixed in 1% OsO4 buffered in 0.05 M phosphate 0.3 M saline at 4 °C for 1 h, subsequently dehydrated in an ascending acetone series followed by propylenoxide and embedded in Araldit. Semi- thin and ultra- thin sections of 1 μm and 70 nm thickness, resp., were cut on a LEICA UC6 ultramicrotome. Semi- thin sections were kept on glass slices and stained with Toluidine blue, while ultra- thin sections were placed on formvar coated, copper single slot grids (1 × 2 mm) and automatically stained with uranyl acetate and lead citrate (QG-3100, Boeckler Instruments). The ultra- thin sections were analyzed in a ZEISS EM10CR transmission electron microscope and documented on phosphor imaging plates (DITABIS) while semi- thin sections were documented using a light microscope (BX-51, Olympus).

### Analysis and 3D reconstruction

Specimens were photographed with a Canon 600D Camera mounted on a Zeiss- Stemi 2000. Serial paraffin and semithin sections were analyzed with an Olympus microscope (BX-51) and photographed with an Olympus camera (Olympus cc12) equipped with the dot slide system (2.2 Olympus, Hamburg) and aligned using Imod [71] and Imod align (http://www.q-terra.de/biowelt/3drekon/guides/imod_first_aid.pdf). 3D- reconstruction of histological sections were performed using Fiji (1.45b) [72]/ trakem [73] and Amira (5.0). 3D reconstructions and volume renderings of the μCT scans were performed using Amira 5.0.

### Data repository and voucher material

The voucher material of *Owenia fusiformis* is deposited at the Institute of Evolutionary Biology and Zooecology of the University of Bonn. Sections of *Myriowenia sp.* are deposited in the Australian Museum, Sydney and curated by Stephen Keable. For data transparency, all aligned serial sections, as well as μCt-scans are freely available in MorphDbase: www.morphdbase.de [74, 75].

*Myriowenia* sp.: www.morphdbase.de/?P_Beckers_20170310-M-96.1

*Owenia fusiformis* (Azan): www.morphdbase.de/?P_Beckers_20170310-M-94.1

*Owenia fusiformis* (Palmgrens silver): www.morphdbase.de/?P_Beckers_20180828-M-104.1

*Owenia fusiformis* (μCT): part 1: www.morphdbase.de/?P_Beckers_20180524-M-103.1. part 2: www.morphdbase.de/?P_Beckers_20180524-M-102.1

## Additional file

Additional file 1: **Figure S1**: Histology Orrhage’s *Owenia fusiformis*. **A**: slide showing sections of *Owenia fusiformis*. **B**: Intermediate filaments (*if*) cross the neuropil of the brain (*br*). The *ecm* of the epidermis is less prominent where the neuropil layer is above it. **C**: Posterior part of the brain (*br*). *if*: intermediate filaments. (JPG 10649 kb)
