# Supplementary figures and images for: The central nervous system of Oweniidae (Annelida) and its implications for the structure of the ancestral annelid brain
Source: Front Zool. 2019 Mar 12;16:6. doi: 10.1186/s12983-019-0305-1 (PMC6417257; doi:10.1186/s12983-019-0305-1)

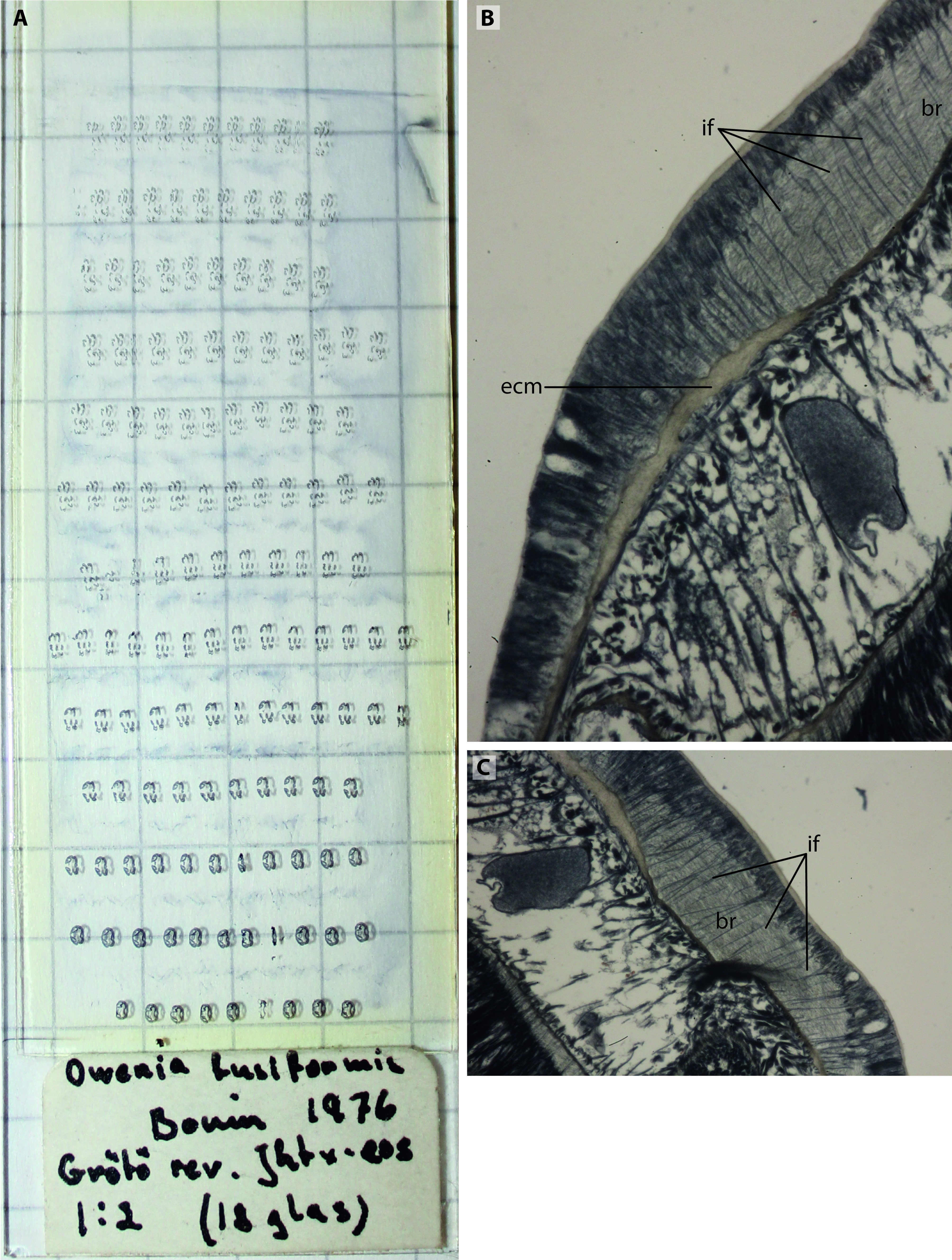

Supplement: Supplementary file 1 — Figure S1: Histology Orrhage’s Owenia fusiformis. A: slide showing sections of Owenia fusiformis. B: Intermediate filaments (if) cross the neuropil of the brain (br). The ecm of the epidermis is less prominent where the neuropil layer is above it. C: Posterior part of the brain (br). if: intermediate filaments. (JPG 10649 kb) [file 12983_2019_305_MOESM1_ESM.jpg]
